# Fibronectin synthesis, but not α-smooth muscle expression, is regulated by periostin in gingival healing through FAK/JNK signaling

**DOI:** 10.1038/s41598-018-35805-6

**Published:** 2019-02-25

**Authors:** Shawna S. Kim, Georgia E. Nikoloudaki, Sarah Michelsons, Kendal Creber, Douglas W. Hamilton

**Affiliations:** 1 Department of Anatomy & Cell Biology, Schulich School of Medicine and Dentistry, The University of Western Ontario, London, Ontario N6A 5C1 Canada; 2 Department of Biomedical Engineering, Schulich School of Medicine and Dentistry, The University of Western Ontario, London, Ontario N6A 5C1 Canada; 3 Division of Oral Biology, Schulich School of Medicine and Dentistry, The University of Western Ontario, London, Ontario N6A 5C1 Canada; 4 Dentistry, Schulich School of Medicine and Dentistry, The University of Western Ontario, London, Ontario N6A 5C1 Canada

## Abstract

During skin healing, periostin facilitates myofibroblast differentiation through a β1 integrin/FAK dependent mechanism and continued expression is associated with scarring. In contrast to skin, gingival tissue does not typically scar upon injury, but the role of periostin in gingival healing has never been investigated. Using a rat gingivectomy model, we show that the gingival architecture is re-established within 14 days of wounding. Periostin mRNA levels peak at day 7 post-wounding, with persistence of periostin protein in the connective tissue through day 14. Collagen type I and lysyl oxidase mRNA levels peak at day 7 post wounding, which corresponded with the peak of fibroblast proliferation. Although α-smooth muscle actin mRNA levels increased 200-fold in the tissue, no myofibroblasts were detected in the regenerating tissue. *In vitro*, human gingival fibroblast adhesion on periostin, but not collagen, was inhibited by blocking β1 integrins. Fibroblasts cultured on periostin exhibited similar rates of proliferation and myofibroblast differentiation to cells cultured on collagen only. However, human gingival fibroblasts cultured in the presence of periostin exhibited significantly increased fibronectin and collagen mRNA levels. Increases in fibronectin production were attenuated by pharmacological inhibition of FAK and JNK signaling in human gingival fibroblasts. *In vivo*, mRNA levels for fibronectin peaked at day 3 and 7 post wounding, with protein immunoreactivity highest at day 7, suggesting periostin is a modulator of fibronectin production during gingival healing.

## Introduction

Periostin is a non-structural secreted matricellular protein that is highly expressed in collagen-rich tissues^1^, with its role in tissue development, repair, and remodeling becoming increasingly determined. Although knockout mice are viable, periostin deletion manifests in disruption of several collagenous-based tissues, particularly in those subject to constant mechanical loading^2^. During development, periostin has shown to play a pivotal role in several processes related to determination of cell fate^3^, including regulating the differentiation of mesenchymal cushion progenitor cells in the heart to contractile myofibroblasts^4^. Although periostin is considered a non-structural ECM component, it has been shown to modulate cross-linking and stabilization of the extracellular matrix, including collagen fibrillogenesis^5,6^ as well as the incorporation of tenascin-C^7^ and BMP-1^8^ into the extracellular matrix. Moreover, periostin is able to interact with fibronectin type I, type V collagen, BMP-1, as well as other periostin molecules^7,9^. This demonstrates a critical role for periostin in extracellular matrix (ECM) homeostasis and the regulation of cell phenotype.

Following acute injury, periostin is up-regulated in various tissues including bone^10^, heart^6,11^, vasculature^12,13^, muscle^14^, and skin^15,16^. Successful healing of soft tissues such as skin follows a precise and predictable series of overlapping events encompassing inflammation, proliferation, re-epithelialization, and matrix remodeling^17^. This results in re-establishment of the epithelium and underlying connective tissue restoring barrier function. After inflammation, as granulation tissue is secreted, mesenchymal cells including fibroblasts migrate into the wound site where they proliferate and synthesize provisional ECM, which is subsequently remodeled to regain the integrity of the connective tissue^18,19^. In skin, periostin is required to modulate alpha-smooth muscle actin (α-SMA) expression in fibroblasts, which contract the wound edges together and in conjunction with re-epithelialization, facilitate wound closure^15^. Periostin expression coincides with the proliferative and remodeling phases of repair in skin^15,20,21^ and is induced by transforming growth factor-beta (TGF-β). With the use of knockout mice, periostin has been shown to regulate collagen synthesis^6^, myofibroblast differentiation^15^, and fibroblast proliferation^22^ during healing. Linked strongly to different pro-fibrotic events in the proliferative phase of skin healing, prolonged expression of periostin is associated with hypertrophic and keloid scarring. Scarring in skin is characterized by the persistence of myofibroblasts, cells expressing α-SMA, which our lab has shown is modulated by periostin through β1 integrin and focal adhesion kinase (FAK) dependent manner^15^.

Similar to skin in structure and organization, gingival tissue interfaces with teeth, preventing bacterial infiltration into the tooth sockets and alveolar bone. Gingival tissue however, heals with significantly less scarring when compared to skin, with many similarities evident to the healing of fetal tissue^23–26^. Gingival tissue has been shown to have reduced levels of TGF-β mRNA and protein and exhibits reduced wound contraction compared to skin in pig models^27^. Interestingly, gingiva wounds heal faster than in skin even in the same animal. There is considerable evidence showing that oral fibroblasts are inherently phenotypically different from dermal fibroblasts. Oral fibroblasts are less responsive to TGF-β1 stimulation, which results in less α-SMA expression compared to dermal fibroblasts *in vitro*^28,29^. Studies have also shown that gingival fibroblasts exhibit less of a pro-fibrotic or scarring phenotype in three-dimensional culture; increased synthesis of levels of genes associated with ECM remodeling and inflammation^30^. Of potential significance, gingival fibroblasts were less contractile compared to skin fibroblasts, a process we have shown in murine skin is modulated by periostin^15^. However, whether periostin is upregulated in gingival tissue healing has never been investigated.

We hypothesized that periostin would be transiently upregulated following gingival wounding, where it would modulate fibroblast differentiation and matrix synthesis. Using a rat gingivectomy model, we show periostin expression is upregulated at day 3, peaking at day 14. In contrast to skin, no α-SMA myofibroblasts are present during gingival healing. Using human recombinant periostin, we demonstrate that gingival fibroblasts increase fibronectin and collagen synthesis, but no effects of periostin on myofibroblast differentiation or proliferation were observed. We conclude that periostin regulates extracellular matrix synthesis during gingival healing.

## Results

### Gingival tissue is re-established within 14 days of gingivectomy

**Figure 1:**
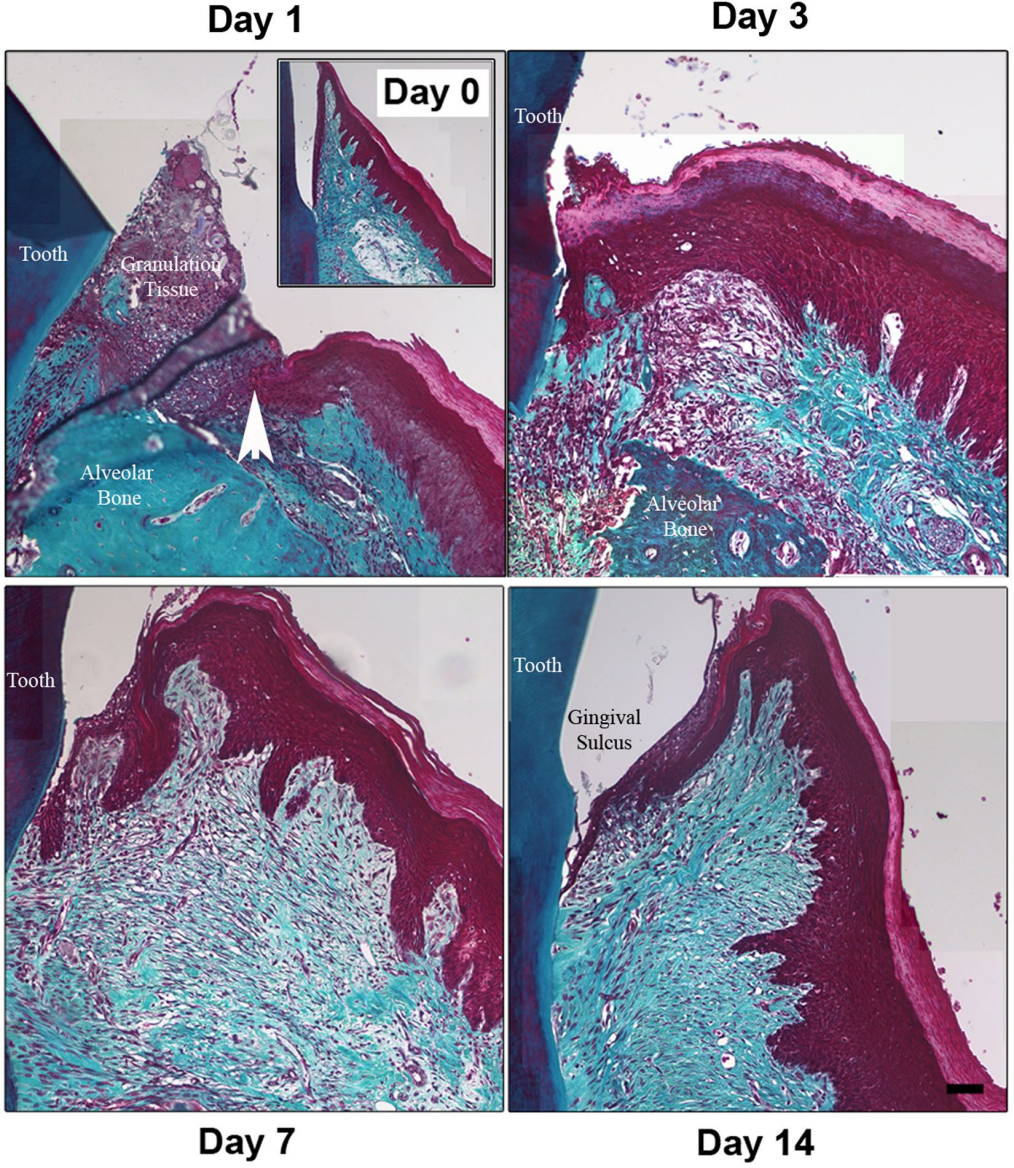
Gingival architecture is re-established 14 days post gingivectomy. Gingivectomy was performed in rats. (**A**) Histological sections of gingiva from rats at 1, 3, 7, and 14 days post-wounding and unwounded gingiva (day 0) were stained using Masson’s trichrome. The images were taken at 20x magnification and stitched together for each tissue. Wound edge is denoted by white arrowhead. *Scale bar*, 50 μm.

To investigate the gingival healing process temporally, we assessed the regeneration of the gingival connective tissue and oral epithelium in rats at days 1, 3, 7, and 14 post-wounding, with unwounded gingiva serving as a structural baseline control (day 0) (Fig. 1). Using Masson’s trichrome staining which allows visualization and differentiation of fine and coarse collagen fibers that appear in the remodeling phase of wound healing^31^, the normal unwounded gingiva contained dense bundles of collagen in the connective tissue, with numerous blood vessels evident (Fig. 1). At day 1 post wounding, granulation tissue filled the defect between the tooth surface, the alveolar bone and the site of injury. 3-days post wounding, re-epithelialization of the defect had occurred, although the normal sulcular gingival epithelial structure was absent. Beneath the epithelium, regeneration of the gingival connective tissue was evident although collagen deposition was minimal (Fig. 1). At 7 days post-wounding, fine collagen fibers were observed as gingival tissue began regenerating. Coarse, more densely organized collagen fibers are also found at the proximity of the initial wound edge in the connective tissue at 7 days. At 14 days post-wounding, dense accumulation of coarse collagen fibers was evident in the regenerated tissue (Fig. 1), with a structure and density similar to unwounded tissue. Furthermore, the normal sulcular gingival structure was re-established by day 14.

### Periostin is upregulated in gingival healing

**Figure 2:**
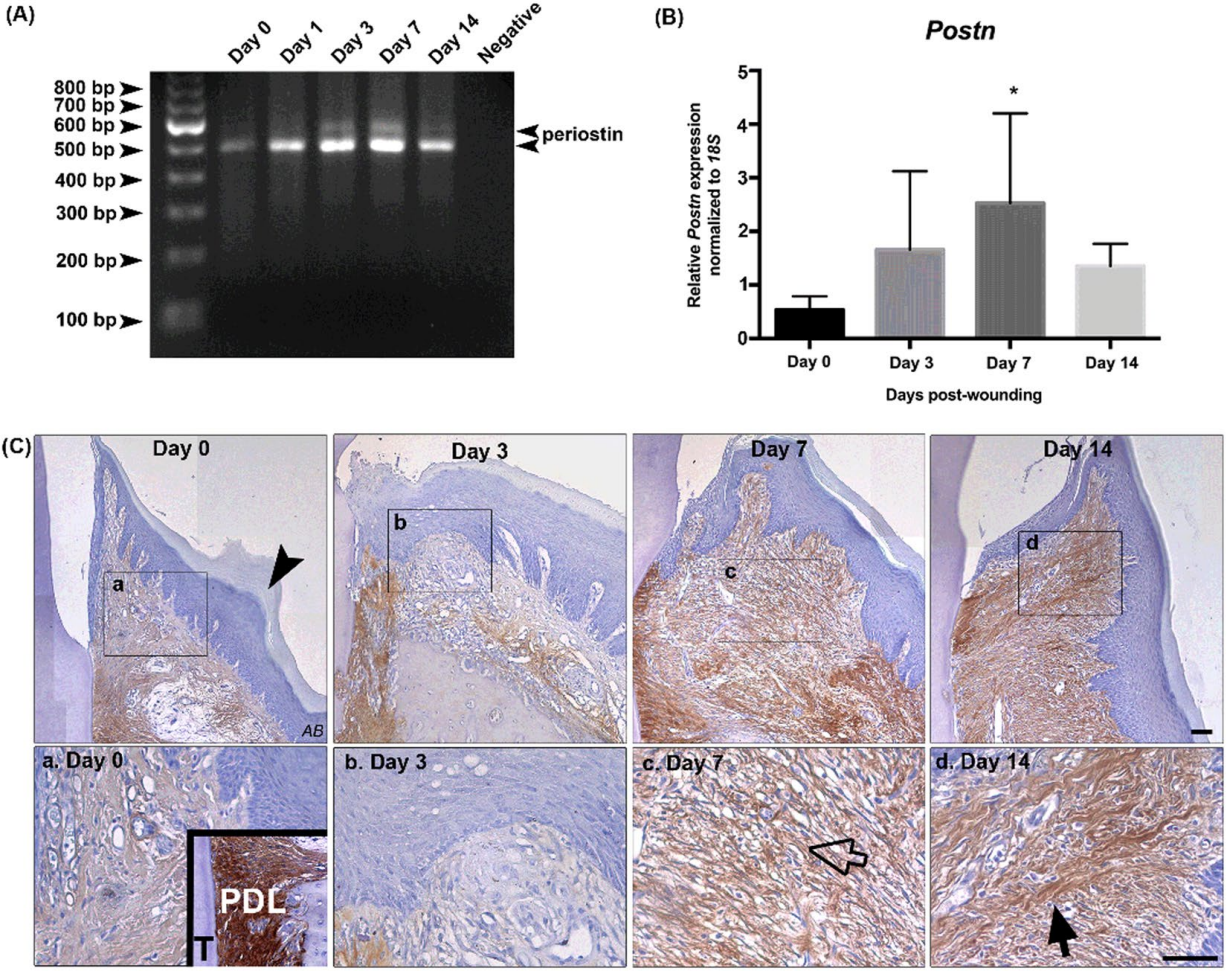
Periostin increases in gingival healing following gingivectomy. (**A**) Gingival tissues dissected from rats were assessed for *POSTN* gene expression. Isoform analysis using RT-PCR demonstrated the presence of 2 isoforms of *POSTN*. (**B**) Analysis of the wild-type isoform of *POSTN* using Taqman PCR showed periostin mRNA peaked at day 7 post wounding. Data represents mean fold gene expressions ± s.d. relative to control day 0 of 3 independent rats. Data was analyzed via one-way ANOVA (*p < 0.05). (**C**) Representative images of immunoreactivity for periostin of gingiva at 1, 3, 7, and 14 days post-wounding and unwounded gingiva (day 0). The inset of day 0 demonstrates periostin positive periodontal ligament (*PDL*), between the tooth (*T*) and the alveolar bone (*AB*). Regions of rectangular boxes are shown in (**a**–**d**) *Scale bar*, 50 μm.

We next quantified the temporal expression of periostin during gingival healing. To screen for different periostin splice variants, total mRNA was reverse transcribed by RT-PCR. We chose a PCR amplification strategy covering the isoform cassette from exon 16 to exon 23 because previously described isoforms in rats exhibited splice alteration in the C-terminal region. 2 bands of PCR products were revealed at days 3 and 7 on the acrylamide gel (Fig. 2A). One band corresponded to full-length isoform and the second band, which was more prominent, to a truncated 500 bp size. Using Taqman PCR, the full length periostin transcript was significantly increased at day 7 post-wounding (*p* < 0.05) (Fig. 2B). To evaluate periostin protein expression in gingival wound healing, tissue sections from rats at days 3, 7, and 14 post-wounding, with unwounded gingiva serving as a baseline control (day 0) were stained for periostin using immunohistochemistry (Fig. 2C,a). In the normal unwounded gingiva, periostin immunoreactivity was weakly detected in the ECM of the connective tissue, with increased periostin levels evident in the periodontal ligament as has been previously described^2,32–34^. At 3 days post-wounding, periostin immunoreactivity was reduced within the granulation tissue and surrounding the lesion (Fig. 2C,b). Furthermore, upregulation of periostin was not evident in the basement membrane under the proliferating and migrating oral epithelial cells as we have shown in skin^21^. Periostin immunoreactivity was the highest at days 7 and 14 post wounding, which corresponded with tissue regeneration and collagen deposition in the connective tissue underlying the oral epithelium (Fig. 2C,c,d). Periostin was detected throughout the extracellular area of maturing granulation tissue at day 7 (Fig. 2C,c). Immunoreactivity also increased at the proximity of the initial wound edge at 7 days. At day 14, periostin labeling was denser than baseline healthy tissue, but was distributed in a similar manner throughout the connective tissue (Fig. 2C,d). Immunoreactivity for periostin was not detected in the oral epithelium or basement membrane.

### A biphasic proliferative response is present in gingival healing

**Figure 3:**
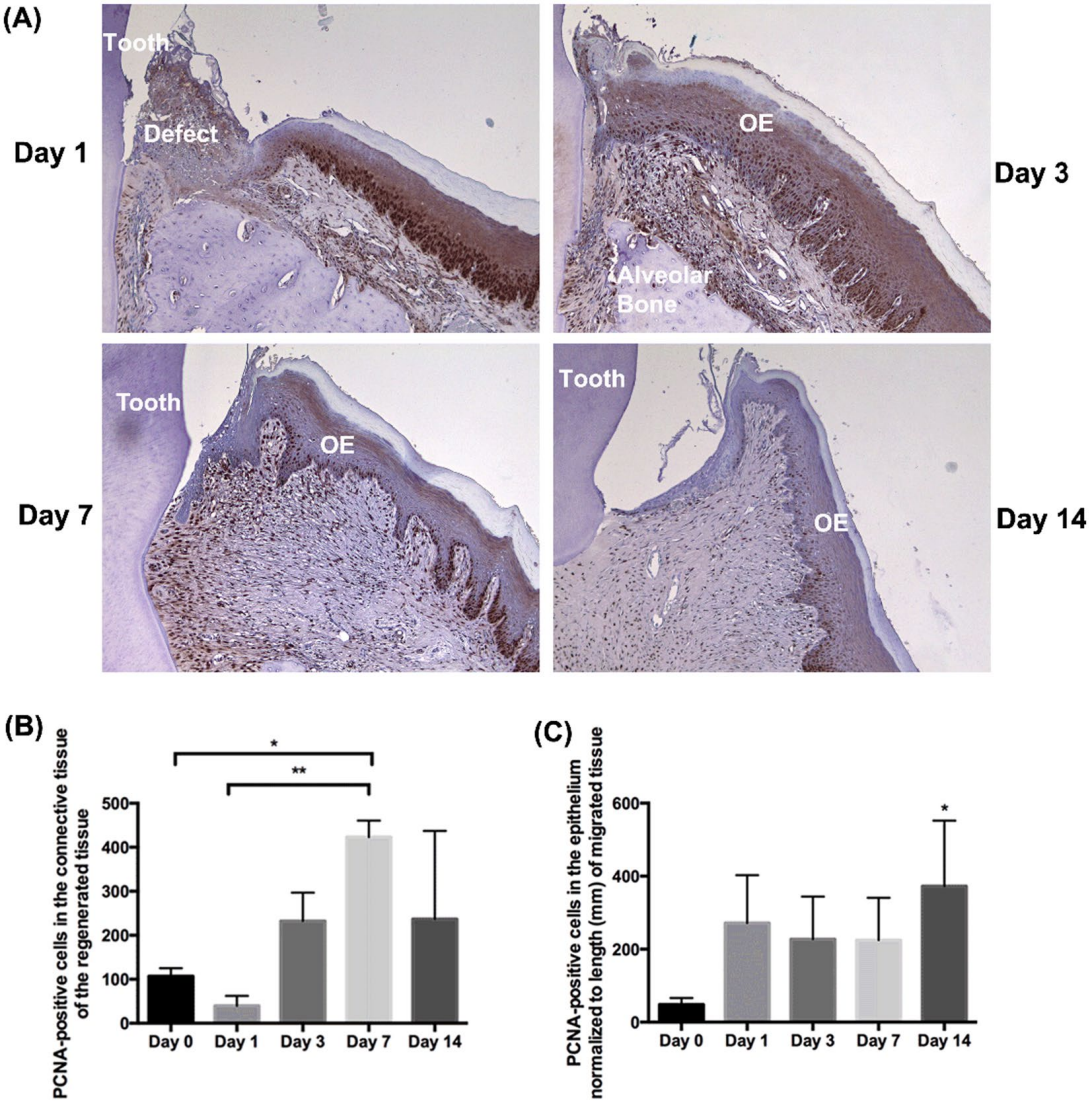
Cell proliferation in gingival healing following gingivectomy *in vivo*. Gingivectomy was performed in rats. (**A**) Representative images of immunoreactivity for PCNA in gingiva at 1, 3, 7, and 14  days post-wounding. (**B**) Quantification of PCNA^+^ cells in the connective tissues of gingival sections from unwounded rat (day 0), and rats at 1, 3, 7, and 14 days post-wounding. Data represents mean PCNA^+^ cells ± s.d. of 3 rats. (**C**) Quantification of PCNA^+^ epithelial cells normalized to the length of the re-epithelized tissue of gingival sections from unwounded rat (day 0), and rats at 1, 3, 7, and 14 days post-wounding. Data represents number of PCNA^+^ cells normalized to the corresponding length of re-epithelized tissue (mm) ± s.d. of 3 rats. Data was analyzed via one-way ANOVA (*p < 0.05 and **p < 0.01 relative to day 0 and day 1, respectively).

To quantify proliferation during gingival wound healing, tissues were labeled with proliferating cell nuclear antigen (PCNA) at days 1, 3, 7, and 14 post-wounding and compared to unwounded gingiva as a baseline (Fig. 3). In the unwounded gingiva, PCNA positive (PCNA^+^) cells were only sparsely observed in both the connective tissue and the epithelium. At day 1 post-wounding, PCNA^+^ oral epithelial cells were observed (Fig. 3A). At day 3, PCNA^+^ cells were evident throughout the epithelium and the connective tissue. At day 7, which correlates with matrix deposition in the connective tissue under the oral epithelium, PCNA^+^ cells were observed in the connective tissue and also in the epithelium. At day 14, PCNA^+^ cells were more sparsely distributed in the connective tissue and the epithelium, at similar levels to normal tissue. Significantly greater numbers of PCNA^+^ cells were evident in the connective tissue at day 7, compared to day 0 (*p* < 0.05) and day 1 (*p* < 0.01) (Fig. 3B). PCNA^+^ epithelial cells (normalized to the corresponding length of the re-epithelialized tissue) started to increase at day 1 post-wounding although not significant (Fig. 3C). At day 14, PCNA^+^ epithelial cells were significantly increased compared to day 0 tissue (*p* < 0.05).

### Myofibroblasts are not present in gingival healing

**Figure 4:**
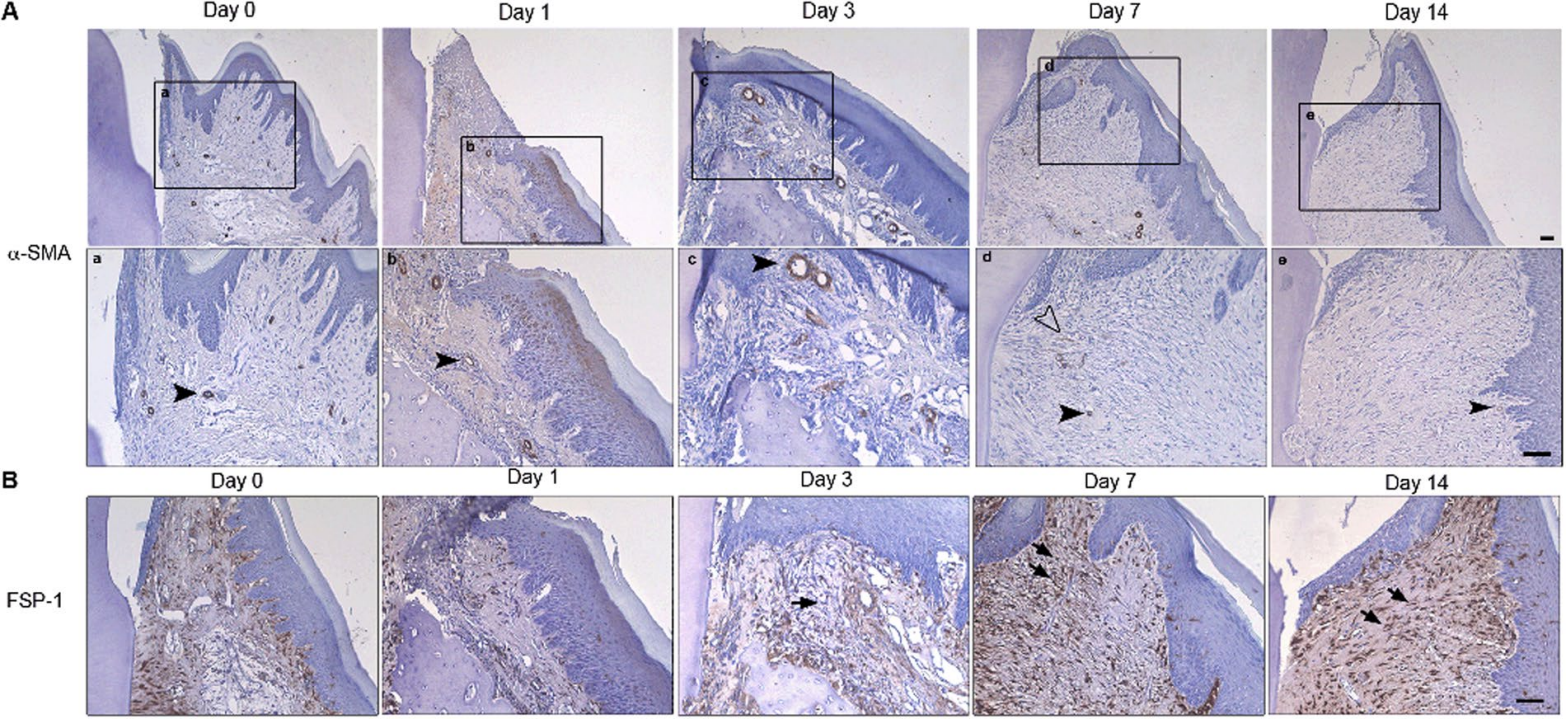
α-SMA is not expressed at the protein level following gingivectomy *in vivo*. (**A**) Representative images of immunoreactivity for α-SMA of gingiva at 1, 3, 7, and 14 days post-wounding and unwounded gingiva (day 0). Histological sections from rats after 1, 3, 7, and 14 days post-wounding and unwound gingiva were incubated with a primary antibody against α-SMA. The primary antibody was detected by using peroxidase-conjugate secondary antibody and DAB. All sections were counterstained with haematoxylin. Panels (a–e) are higher magnifications of the corresponding regions indicated by rectangular boxes. Black arrowheads denote α-SMA-positive blood vessels. Few myofibroblasts were evident at day 7 indicated by hollow arrowhead. (**B**) Representative images of immunoreactivity for FSP-1 of gingiva following gingivectomy. Anti-FSP-1 was detected by using peroxidase-conjugate secondary antibody and DAB, and the sections were counterstained with haematoxylin. FSP-1-positive cells in the regenerated connective tissue are denoted by black arrows. *Scale bar*, 50 μm.

To evaluate whether myofibroblasts were present during gingival wound healing, immunoreactivity for α-SMA was assessed in gingival tissue sections from rats at days 1, 3, 7, and 14 post-wounding and unwounded gingiva (day 0). In all tissues at all time points investigated, immunoreactivity for α-SMA was only evident in the vasculature, with sparse myofibroblasts detected at day 7 in connective tissue close to the tooth (Fig. 4A). To assess fibroblast localization during gingival healing, histological sections were stained with the antibody against fibroblast specific protein-1 (FSP-1) (Fig. 4B). In normal gingiva, FSP-1-positive cells were evident throughout the connective tissues. At day 3, immunoreactivity for FSP-1 was significantly reduced. At days 7 and 14, cells positive for FSP-1 were elevated in the regenerating tissue. This was however higher than evident in unwounded tissue, due to an increase in fibroblast density.

### Genes associated with collagen remodeling are significantly increased in the proliferative phase of healing

**Figure 5:**
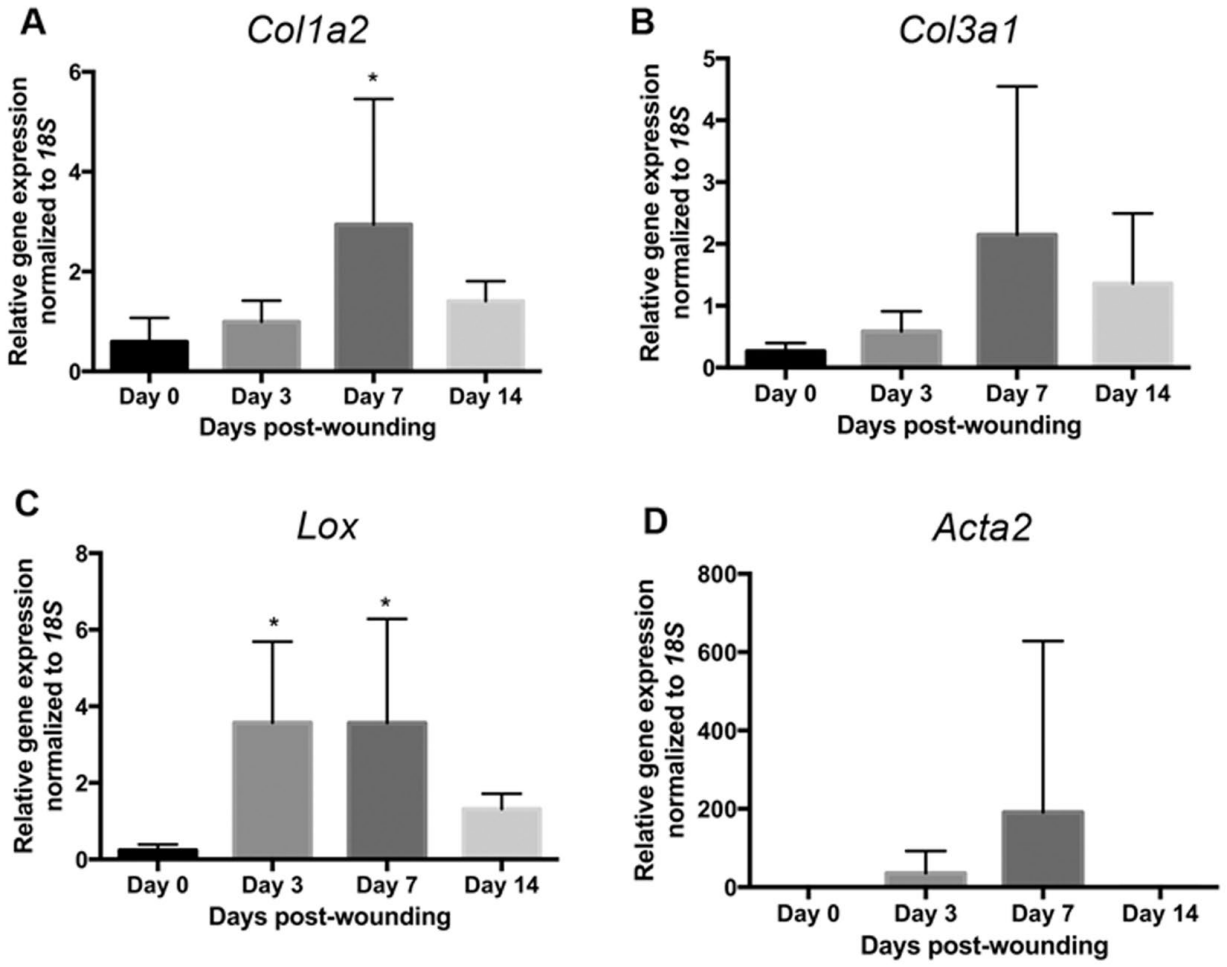
Quantification of Matrix Associated gene expression of *Col1a2*, *Col3a1*, *Lox*, *Acta2*, and *Fn1*. Gingival tissues were dissected from the rats to assess for gene expressions of **(A)** *Col1a2*, **(B)** *Col3a1*, **(C)** *Lox*, **(D)** *and Acta2*. Target gene expressions were normalized to *18* *S* using the ΔΔCt method. Data represents mean fold gene expressions ± s.d. relative to control day 0 of 6 independent animals run in triplicate. Data was analyzed via one-way ANOVA (*p < 0.05; **p < 0.01).

We next assessed the expression for genes associated with healing; specifically the fibrillar collagens I (*Col1a2)*, III (*Col3a1)*, the collagen cross-linking enzyme lysyl oxidase (*Lox*), and α-SMA (myofibroblast differentiation) during gingival wound healing *in vivo*. *Col1a2* mRNA levels peaked at day 7, although *Col3a1* levels at day 7 was not significantly different from unwounded tissue (Fig. 5A,B). *Lox* was significantly higher at both days 3 and 7 (Fig. 5C). While *Acta2* message levels increased, statistically significant differences were not observed (Fig. 5D).

### Recombinant periostin does not alter HGF proliferation or induce myofibroblast differentiation in HGFs

**Figure 6:**
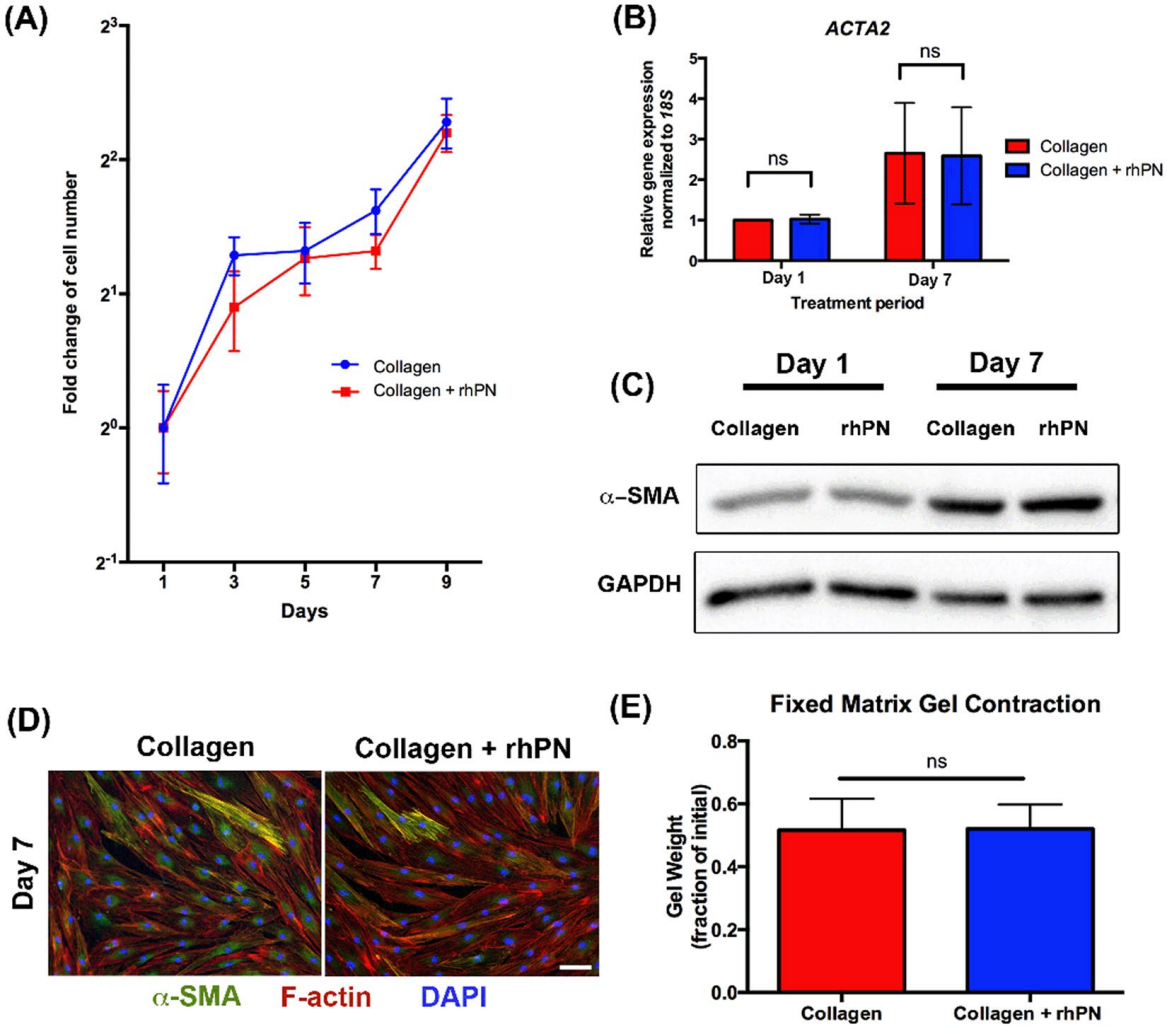
rhPN does not affect HGF proliferation nor modulate myofibroblast differentiation *in vitro*. (**A**) HGFs cultured on collagen or collagen + rhPN coated plates for 1, 3, 5, 7, and 9 days were assessed for proliferation using CyQUANT assay kit to determine DNA contents. A standard curve was used to extrapolate cell number. Data represents fold cell number increase ± s.d. relative to day 1. Data was analyzed via two-way ANOVA d (*p* > 0.05). (**B**) HGFs cultured on collagen or collagen + rhPN coated plates for 1 day and 7 days were assessed for gene expressions of *ACTA2*. Target gene expression was normalized to *18* *S* using the ΔΔCt method. Data represents mean fold gene expressions ± s.d. relative to control day 1 (collagen alone) of 3 independent experiments in triplicates. Data was analyzed via Student’s t-test (unpaired) within each time-point. (**C**) Western blot was used to assess α-SMA protein level of HGFs cultured on collagen or collagen + rhPN coated plates. GAPDH was used as a loading control. (**D**) Immunocytochemistry was performed with HGFs cultured on collagen or collagen + rhPN coated plates for 7 days to visualize myofibroblasts. Representative fluorescent images of HGFs labeled for α-SMA (green), F-actin (red), and nuclei (blue). Myofibroblasts were revealed by α-SMA-positive stress-fibers. *Scale bar*, 50 μm. (**E**) Fixed collagen gel contraction assay of HGFs in the absence and presence of rhPN (5 μg/ml). Gel contraction was quantified by loss of gel weight, compared with gels lacking cells. Data was analyzed via Student’s t-test (unpaired) within each time-point (*p* > 0.05; ns, not significant).

To examine the functional role of periostin in gingival healing, we utilized *in vitro* assays using our established HGF model system. Specifically, we investigated if recombinant periostin (rhPN) influenced the proliferation rate or myofibroblast differentiation of HGFs. HGFs cultured on collagen alone and collagen with rhPN exhibited a 5-fold increase in cell number by day 9 (Fig. 6A) with no significant difference in cell number evident between the conditions (*p* > 0.05). Although no myofibroblast differentiation was evident during gingival healing in rats, periostin is known to modulate expression of α-SMA in skin healing^15^. Therefore, we first assessed whether rhPN influenced α-SMA levels using RT-qPCR, western blot, and collagen gel contraction assays. HGFs cultured on collagen with rhPN showed no difference in *ACTA2* mRNA levels at 1 day and 7-days post seeding compared to HGFs cultured on collagen alone (*p* > 0.05) (Fig. 6B). Similarly, no difference in α-SMA protein level in HGFs cultured for 1 day and 7 days was observed between conditions (Fig. 6C). Immunocytochemistry showed no difference in the ability of the HGFs to differentiate into myofibroblasts, indicated by α-SMA-incorporation into stress fibers (Fig. 6D). Quantification of contraction via measurement of gel weight demonstrated that rhPN did not increase HGF-mediated contraction of a collagen gel matrix (*p* > 0.05) (Fig. 6E).

### Recombinant human periostin increases fibronectin synthesis in HGFs *in vitro*

**Figure 7:**
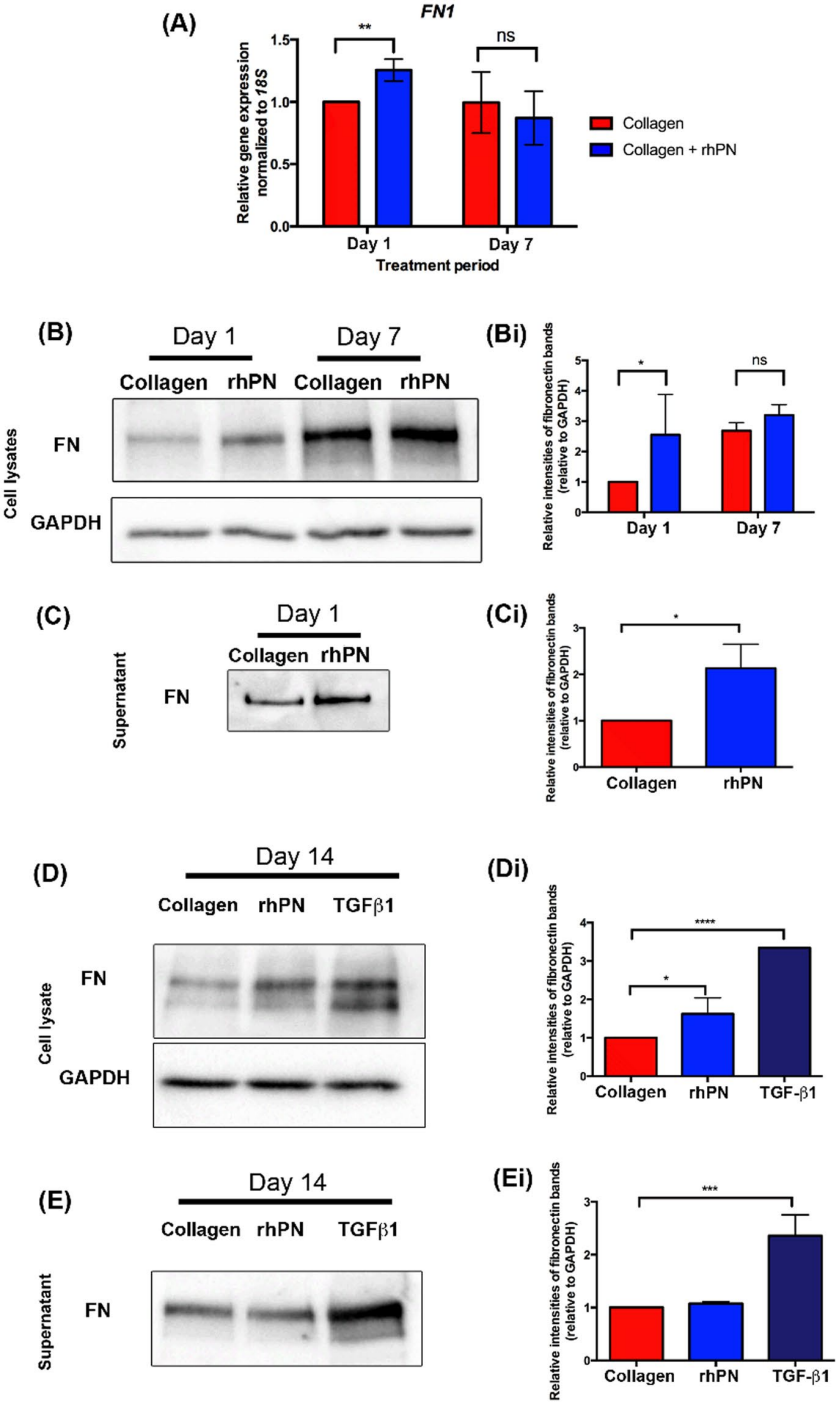
HGFs cultured with rhPN exhibit increased fibronectin synthesis. (**A**) HGFs cultured on collagen or collagen + rhPN coated plates for 1 day and 7 days were assessed for gene expressions of *FN1*. Target gene expression was normalized to *18* *S* using the ΔΔCt method. Data represents mean fold gene expressions ± s.d. relative to control day 1 (on collagen alone) of 3 independent experiments in triplicates. Data was analyzed via Student’s t-test (unpaired) within each time-point (**p < 0.01; ns, not significant). (**B**) Fibronectin (FN) protein levels were assessed using western blots in cell lysates when HGFs are cultured on collagen (Col) or collagen + rhPN (rhPN) for 1 day and 7 days. GAPDH was used as a loading control. (**C**) Fibronectin protein detection in the supernatants from HGFs cultured on Col or rhPN for 1 day using western blot. (**D**,**E**) HGFs cultured on Col or rhPN coated plates for 14 days were assessed for fibronectin protein level. TGF-β1 treatment of HGFs on collagen-coated plates served as a positive control. (**D**) Fibronectin protein levels were assessed using western blots in cell lysates (**D**) when HGFs are cultured for 14 days and in supernatant (**E**) obtained from the last day of the culture. GAPDH was used as a loading control for cell lysates. **Bi**,**Ci**,**Di**,**Ei**. Densitometry analyses of the fibronectin bands were performed. For proteins from cell lysates, fibronectin bands were normalized to corresponding GAPDH bands. Data represents mean fold band intensities ± s.d. relative to control day 1 (collagen alone) of 3 independent experiments in triplicates. Data was analyzed via Student’s t-test (unpaired) within each time-point (*p < 0.05; ns, not significant) (**Bi-Ci**) and one-way ANOVA (*p < 0.05, ***p < 0.001, ****p < 0.0001) (**Di-Ei**).

As periostin did not influence proliferation or myofibroblast differentiation which confirmed our *in vivo* findings, we next cultured HGFs on collagen and collagen with rhPN coated-plates and assessed collagen and fibronectin levels. Fibronectin synthesis by HGFs cultured on collagen alone and collagen with rhPN coated-plates, was assessed using RT-qPCR and western blotting. *FN1* mRNA levels in HGFs cultured with rhPN was significantly higher compared to cells on collagen alone, at day 1 (Fig. 7A) (p < 0.05). At 7 days, no significant difference in *FN1* mRNA levels were measured (*p* > 0.05). Similarly, western blots demonstrated that fibronectin protein levels were greater in cell lysates cultured with rhPN compared to cells cultured with collagen alone at 1 day (Fig. 7B), which was confirmed with densitometric analysis of fibronectin bands normalized to corresponding GAPDH band intensities (*p* < 0.05) (Fig. 7Bi). Fibronectin levels in the supernatant from cells cultured with rhPN were also increased compared to the supernatant from cells on collagen alone at day 1 (Fig. 7C, Ci). HGFs cultured with rhPN for 14 days demonstrated greater fibronectin protein levels in the cell lysates (*p* < 0.05) (Fig. 7D, Di) but not in the supernatants at the end of the culture (*p* > 0.05) (Fig. 7E, Ei). HGFs cultured with TGF-β1, which served as a positive control, resulted in increases of fibronectin in the cell lysates and the supernatants at 14 days (*p* < 0.05) (Fig. 7Di, Ei).

### Recombinant human periostin increases collagen synthesis in  HGFs *in vitro*

Quantification of *COL1A2* and *COL3A1* mRNA levels by RT-qPCR demonstrated that HGFs on rhPN had significantly higher mRNA levels compared to HGFs on collagen alone at day 1 (Supplementary Fig. 3A) (*p* < 0.05). At 7 days, no significant differences were observed in mRNA levels between controls and HGFs cultured on rhPN (*p* > 0.05). To assess collagen at the protein level, hydroxyproline assays were performed on HGFs cell lysates and media obtained at the end of 14 days cultures (Supplementary Fig. 3B). Hydroxyproline levels in cell lysates cultured with rhPN or TGF-β1 were significantly greater than cells cultured on collagen alone (*p* < 0.05). Hydroxyproline content in the media samples from cells cultured with rhPN or TGF-β1 were also significantly greater than the supernatant from control cells (*p* < 0.05).

### Periostin mediates fibronectin synthesis through FAK and JNK

**Figure 8:**
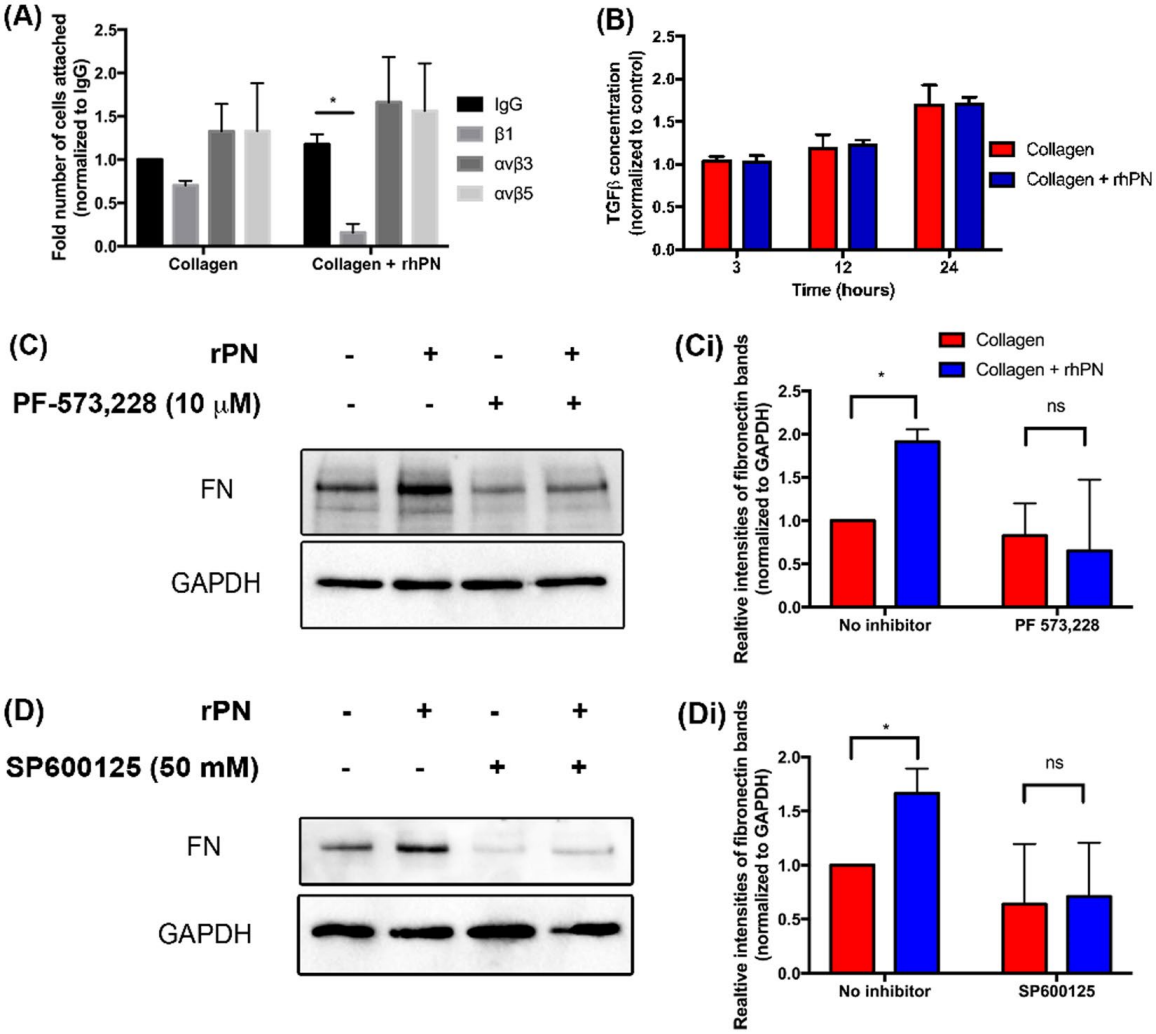
Periostin mediates fibronectin synthesis through integrin β1, FAK and JNK. (**A**) Integrin β1, αvβ3, and αvβ5 of HGFs were neutralized using the integrin subunit specific antibodies. Data represents relative number of attached cells after 1 hour of seeding and error bars represent STD. Data was analyzed using two-way ANOVA with bonferonni post-test (*p < 0.05). (**B**) ELISA quantification of TGF-β1 at 3, 12 and 24 hrs post seeding. (**C**,**D**) FN protein levels were assessed using western blots in cell lysates when HGFs are cultured with or without rhPN and/or PF573,228, or SP600125 for 1 day. GAPDH was used as a loading control. **Ci**,**Di** Densitometry analyses of the fibronectin bands were performed. Fibronectin bands were normalized to corresponding GAPDH bands. Data represents mean fold band intensities ± s.d. relative to control (no rhPN) of 3 independent experiments in triplicates. Data was analyzed using one-way ANOVA (*p < 0.05; ns, not significant).

Previous studies have demonstrated that periostin interacts via the tandem FAS1 domains with integrin receptors such as αvβ3, αvβ5, and β1^35–39^. Neutralization of β1, but not αVβ3 and αVβ5 led to a significant reduction in HGF adhesion compared to control IgG neutralized cells on collagen + rhPN (*p < *0.05) (Fig. 8A). Neutralizing integrin subunits β1, αVβ3, or αVβ5 did not significantly change cell attachment on collagen alone (*p* > 0.05).

To further assess how periostin modulates fibronectin synthesis, we first assessed whether culture of HGFs on periostin increased TGF-β1 protein levels, as TGF-β1 has been shown to increase fibronectin synthesis through c-Jun N-terminal kinase JNK^40^. Culture of HGFs on collagen or collagen + rhPN showed no significant differences on TGF-β1 levels (Fig. 8B). We next inhibited FAK and JNK using PF-573,228, and SP600125 respectively to quantify their effects on periostin-mediated fibronectin synthesis. At baseline, HGFs cultured on collagen + rhPN had increased fibronectin production compared to HGFs on collagen alone (*p* < 0.05). When FAK was inhibited, no increase in fibronectin protein levels were observed in HGFs in the presence of rhPN Vs collagen alone (Fig. 8C). Similarly, when JNK was inhibited by SP600125, the band intensity for fibronectin was not significantly different between collagen alone and collagen + rhPN (*p* > 0.05) (Fig. 8D).

### Fibronectin synthesis corresponds to periostin upregulation during gingival healing

**Figure 9:**
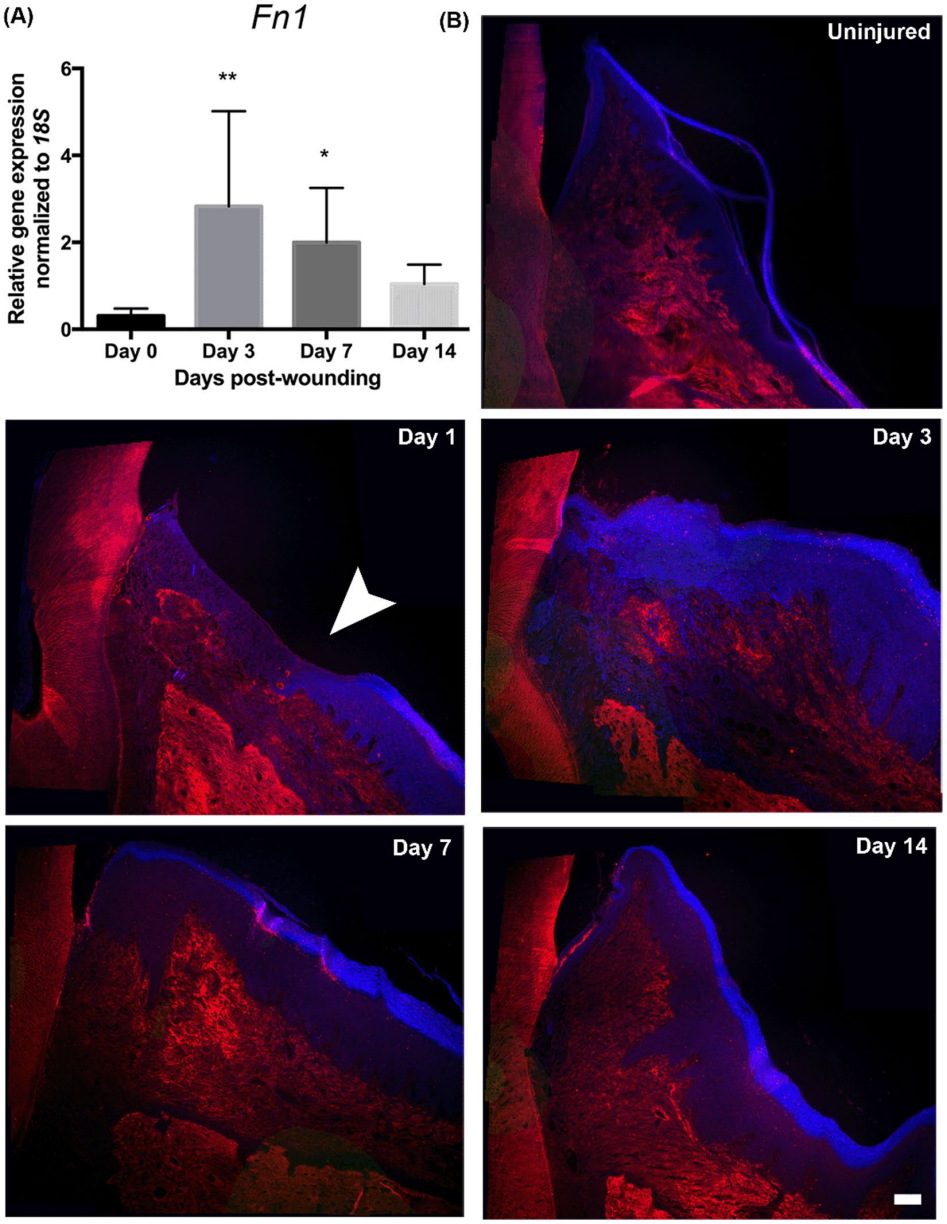
Fibronectin deposition corresponds to periostin protein peak in gingival healing *in vivo*. (**A**) Gingival tissues were dissected from the rats to assess for gene expressions of Fibronectin *(Fn1)*. Gene expression was normalized to *18* *S* using the ΔΔCt method. Data represents mean fold gene expressions ± s.d. relative to control day 1 (on collagen alone) of 3 independent experiments in triplicates. Data was analyzed via one-way ANOVA (*p < 0.05; **p < 0.01). (**B**) Representative images of immunoreactivity for fibronectin of gingiva at 1, 3, 7, and 14 days post-wounding and unwounded gingiva (day 0). Histological sections were incubated with a primary antibody against fibronectin. The primary antibody was detected with Cy5 (red) conjugated secondary antibody. Nuclei are stained with Hoechst 33342 dye (blue). The images were taken at 10x magnification and stitched together for each tissue. Wound edge is denoted by white arrowhead. *Scale bar*, 50 μm.

To evaluate fibronectin in gingival wound healing, we first examined mRNA levels for fibronectin (Fig. 9A). *Fn1* mRNA levels were significantly increased by day 3 post wounding, which was maintained at day 7 before it declined. To assess fibronectin deposition, tissue sections from rats at days 1, 3, 7, and 14 post-wounding, with unwounded gingiva serving as a baseline control (day 0) were stained for fibronectin (Fig. 9B). In the normal unwounded gingiva, fibronectin immunoreactivity was distributed throughout the ECM of the gingival connective tissue. At days 1 and 3, fibronectin deposition was low in the granulation tissue, but by day 7, increased fibronectin immunoreactivity in the matrix was observed, which was reduced by day 14. This pattern of fibronectin immunoreactivity corresponded with periostin immunoreactivity at these time points (Fig. 2A).

## Discussion

Periostin is an important protein in ECM homeostasis and remodeling, with prolonged or increased expression levels associated with fibrosis^41^. Excessive scarring results in altered tissue architecture that affects growth, development, and normal tissue function^42,43^. Oral gingival tissue is known to heal with reduced scarring in contrast to skin^23,44^, but the expression profile and potential roles of periostin in gingival healing have never been investigated. In this report, we confirm that periostin is upregulated in gingival tissue in response to injury.

Previous studies including by our group, have shown that following excisional skin wounding in mice, periostin is upregulated at day 3, levels peak at day 7, after which expression eventually returns to baseline^45^. Periostin has been shown to regulate myofibroblast differentiation, matrix synthesis and re-epithelialization in skin^5,15,21^. In this study, we demonstrate that periostin expression was increased at day 7, with protein levels increased yet further at day 14 in the ECM of the connective tissue. In a similar manner to skin healing, periostin upregulation in gingival tissue coincides with the proliferative and remodeling phases of healing, but not the inflammatory phase. Interestingly, periostin protein levels are not increased at day 3 as is seen in skin healing, highlighting a difference in expression profile between the two tissue types. 2 periostin isoforms were detected at days 3, 7, and 14 in gingival tissue, with the smaller isoform more prominent than the isoform that seem to correspond to the full-length. There is a lack of understanding on types of periostin isoforms in rats. Previously performed sequence analysis demonstrated that the dominating periostin isoform encompasses exons 16, 18, 19, 20, 22, and 23^46^. This isoforms size corresponds to the prominent periostin isoform found in our study. Future studies will focus on the signficance of these two different isoforms present in gingival healing.

Recruitment and proliferation of fibroblasts and epithelial cells is a central event during healing to re-establish barrier function, as well as to facilitate matrix synthesis and tissue remodeling^18^. We show here that the initial proliferative response in gingival healing is in the oral epithelium at day 1 post-wounding, with the initial wound site re-epithelialized by day 3. This rapid induction of proliferation and migration in the oral epithelium allows for rapid re-establishment of barrier function to prevent bacterial infiltration of the sub-gingival tissues. In this study, we showed that periostin immunoreactivity does not localize in the basement membrane of rat gingiva at days 0, 1 and 3 post-wounding in areas where epithelial cells undergo significant proliferation. In our previous studies, we have shown that that kinetics of re-epithelialization of 8 mm excisional skin wounds is not altered by periostin deletion^15^; instead a failure of the wounds to contract requires the epithelium to migrate further. However, during healing of incisional healing, which occurs predominantly by re-epithelialization, periostin is significantly upregulated in the basement membrane at the edge of the wound in the undamaged skin, which correlates with the areas of proliferating keratinocytes. Therefore, we conclude that periostin is unlikely to be influencing proliferation and migration of oral epithelial cells during gingival healing.

PCNA labeling becomes evident in fibroblasts in the developing granulation tissue at day 3, with the highest levels of PCNA positive fibroblasts observed in the regenerating tissue at days 7 and 14. As is seen with oral epithelial cells, fibroblast proliferation occurs earlier in the healing response than induction of periostin expression. *In vitro*, HGFs cultured in the presence of rhPN show proliferation rates compared to cells on collagen alone, demonstrating that periostin does not appear to affect proliferation. This is in agreement with our previous findings; periostin deletion in mice does not alter cell number in excisional skin healing and dermal fibroblasts isolated from periostin null mice showed no significant difference in proliferation kinetics compared to wild-type cells^15^. However, Ontsuka and colleagues demonstrated that periostin KO dermal fibroblasts proliferated less, an observation that could be reversed by forced expression or the addition of recombinant periostin^22^. However, the effect of periostin on proliferation of human dermal fibroblasts was concentration dependent, and the effect of rhPN on murine wild-type cells was less than if periostin was overexpressed in the cells. Combining all these independent results with those from this study show that the exact role of periostin on proliferation is unclear and highly dependent on the experimental conditions.

As myofibroblast differentiation during skin healing mediates contraction of the wound edges and is modulated by periostin in mice^15^, we examined whether myofibroblasts were evident in gingival healing. Myofibroblasts incorporate α-SMA into stress fibers that allows for the contraction of their surrounding matrix. Interestingly, we observed a very low level of myofibroblasts at day 7, suggesting that adoption of a contractile myofibroblast phenotype is not a significant event in gingival wound healing. Interestingly, although myofibroblast were largely absent, periostin was still present in the ECM during wound healing, at day 7 and day 14. *In vitro* studies demonstrated that rhPN does not increase α-SMA protein, α-SMA incorporation into stress fibers, nor induce gel contraction. Therefore we conclude that periostin does not induce a contractile myofibroblast phenotype in HGFs as it does in dermal fibroblasts. Behavioral differences between oral and dermal fibroblasts *in vitro* have been reported in numerous studies^47^. On a functional level, gingival fibroblasts exhibit a reduced fibrotic phenotype when directly compared to dermal fibroblasts, manifesting in lower expressions of genes related to TGF-β signaling, ECM, and cell contractility^30^. We have shown previously that in murine skin, periostin modulates α-SMA expression in a focal adhesion kinase (FAK) and β1 integrin engagement dependent manner^15^. Interestingly, phosphorylation of FAK, which is a central molecule activated in adhesive signaling, is required for induction of α-SMA in response to TGF-β1^48,49^. Recently, Guo *et al*. have demonstrated that the HGFs exhibit fewer, smaller focal adhesions concomitant with lower levels of FAK phosphorylation, compared to human dermal fibroblasts^50^. Therefore, the low level of myofibroblasts present during gingival healing may be in part be due to reduced adhesive signaling which ultimately manifests in a failure to activate α-SMA expression.

Increased matrix stiffness is also a trigger for fibroblast to myofibroblast transition by inducing maturation of focal adhesions^51^, which raises the possibility that healing gingival tissue ECM may not possess the same level of stiffness that granulation tissue has in skin. During healing of excisional wound in murine skin, deletion of periostin does not affect α-SMA expression in high-tension areas at the wound edge, but α-SMA is completely absent in the relatively low-tension granulation tissue, suggesting that periostin modulates myofibroblast differentiation in relatively compliant tissue^15^. However, the presence of periostin in gingival tissue is not sufficient to induce α-SMA, suggesting that the maturity of focal adhesions and a lack of adhesive signaling in HGFs more likely results in an absence of myofibroblasts during healing.

Oral mucosa, like fetal tissue, has been suggested to heal more rapidly with less scarring than skin^24,25^. The persistence of myofibroblasts is accepted to be responsible for scar remodeling and fibrosis in many organs^52^. The lack of myofibroblasts evident in gingival healing is similar to the response of fetal tissue at early gestational age, where an absence of myofibroblasts and scarring occurs in wound healing^53^. An increasing propensity for scarring during wound repair parallels higher levels of myofibroblasts that become observed, which is evident with increasing fetal age^54^, suggesting that phenotypic modulation of wound fibroblasts into myofibroblasts may be involved in scar formation. However, there is evidence in the literature suggesting that gingival fibrosis may not involve myofibroblasts^55–58^. Although we need to further confirm time points between 4 and 6 days, our study suggests that the low level of myofibroblast during repair is likely a significant factor relating to scar-less healing evident in gingival tissue.

We next examined whether periostin modulated matrix production by HGFs. In our study, immunohistochemistry demonstrates that periostin protein levels are highest when collagen density is increasing during gingival wound healing. Supporting this, RT-qPCR and hydroxyproline assay demonstrate that HGFs cultured in the presence of rhPN have increased *COL1A2* and *COL3A1* mRNA levels, with greater hydroxyproline content in cell lysates and supernatants. Fibronectin is a regulator of collagen organization and tissue phenotype^59^ and RT-qPCR and western blot also showed that fibronectin is increased in both cell lysates and the conditioned media. Periostin has binding domains that interact with extracellular matrix components such as collagen and fibronectin to modulate tissue structure and function^7^ and is a critical molecule in several tissue types during wound healing^41^. Previous reports support a role for periostin in collagen synthesis and fibrillogenesis in skin and heart^5,60–62^; periostin null mice demonstrate reduced collagen fibril diameters, concomitant with aberrant collagen I fibrillogenesis and collagen cross-linking in skin^5^. Therefore, our data provides further evidence that periostin modulates matrix synthesis. Although technically challenging, future studies will investigate whether deletion of periostin impacts on collagen and fibronectin synthesis during the proliferative and remodeling phases of gingival wound repair.

We next examined the mechanisms through which periostin modulates fibronectin synthesis. Upon changes to the physical structure of the tissue due to injury, quiescent fibroblasts sense these changes in the matrix through integrin-mediated adhesions and respond accordingly to facilitate healing of the tissue^44^. Previous studies have demonstrated that periostin binds to integrin receptors αvβ3, αvβ5, and β1^35–39^. Our study shows that antibody neutralizion of integrin β1 resulted in a significant reduction in HGF attachment on periostin-coated surfaces. This demonstrates that periostin specifically interacts with integrin β1 to initiate signaling cascade in HGFs. Initial clustering of alpha and beta integrin subunits forms nascent adhesions, which translate into focal complexes by accumulation of tyrosine-phosphorylated residues, and by recruiting various molecules such as FAK^63,64^. FAK is known to then activate downstream signaling molecules such as JNK and ERK1/2^65^. In our study, when we inhibited FAK and JNK independently, periostin-induced fibronectin synthesis was attenuated, showing that FAK and JNK are downstream mediators of the periostin induction of fibronectin expression. Although FAK signaling is not enough to induce myofibroblast differentiation in HGFs, we show that it is sufficient for fibronectin synthesis.

In conclusion, we show that periostin is upregulated in gingival healing, although it is not associated with myofibroblast differentiation or proliferation during gingival regeneration. Based on its role in skin healing, the absence of myofibroblasts is likely an underlying reason for reduced scar formation evident in healing of gingival tissue. The primary influence of periostin appears to be modulation of matrix synthesis during healing in rats, which was confirmed by *in vitro* culture of HGFs with rhPN.

## Materials and Methods

### Rat Gingivectomy

45 female Wistar rats, 8 weeks of age (average weight, 281 grams), five litter-mate rats per litter, were used for wound-healing studies. Animal procedures were conducted in accordance with protocols approved by the University Council on Animal Care at University of Western Ontario. Rats were anesthetized by intraperitoneal injection of Ketamine (75 mg/kg) and Xylazine (10 mg/kg). Gingivectomy was performed on the maxillary palatal gingiva close to the upper molars (Supplementary material Fig. S1). Yardley gingival cord packer (HF-120-G; Hu-Friedy; Chicago, IL) was used to disrupt the junctional epithelium and the connective tissue and tooth interface to raise a full-thickness 1 mm wide gingival flap along the first, second and third molars. A no. 15 scalpel blade was used to excise the soft connective tissue flap. Corneal tweezers (81D40.21; Lee Valley; Ottawa, Ontario) was used to precisely remove the excised soft tissue. In a subset of animals (n = 9), maxillary palatal gingiva close to the right upper molars were left untouched to serve as a day 0 baseline. The animals received 0.5 mg/kg Buprenorphine by subcutaneous injection twice daily for 48 hours post-surgery as an analgesic. Animals were maintained on a standard lab chow powdered food diet and were allowed food and water *ad libitum* for the duration of the experiment. Animals were sacrificed at 1, 3, 7, and 14 days post-wounding (n = 9 at each time-point) by carbon dioxide inhalation. Three groups of litters were subjected to tissue preparation and six groups of litters were preceded to total RNA isolation procedure.

### Tissue Preparation

Post euthanasia, rats were decapitated and the heads fixed in 10% neutral buffered formalin (Sigma Aldrich; St. Louis, MO) for 2 days. The 2 hemi-maxillae were dissected out, and decalcified using Cal-Ex*® Decalcifier (Thermo Fisher Scientific; Waltham, MA) for 2 days. Hemi-maxillae were dehydrated through a graded series of ethanol and paraffin processed and embedded, and sectioned at 5 μm for various staining.

### Immunohistochemistry

Immunohistochemistry was performed as previously described^15,21,34^. In brief, tissues were deparaffinized and immune-labeled using primary antibodies against periostin (sc49480; Santa Cruz Biotechnology; Dallas, TX; 1:100), α-SMA (ab5694; Abcam; Cambridge, MA; 1:400), PCNA (ab2426-1; Abcam; 1:100), and FSP-1 (07-2274; Millipore; Billerica, MA; 1:100). Primary antibodies were detected using the ImmPRESS Reagent Kit Peroxidase (Vector Laboratory; Burlingame, CA) and DAB reagent (Vector Laboratory) following the manufacturer’s instructions. Primary antibody delete was used as experimental control. All sections were counterstained with haematoxylin (Sigma Aldrich). To study collagen levels, deparaffinized histological sections were stained using Masson’s trichrome (University Hospital, London, ON) stain. Images were taken with a DM1000 light microscope (Leica; Concord, Ontario) and Leica Application Suite Software (version 3.8).

### Immunofluorescence

Tissue sections were deparaffinized and permeabilized with 0.1% Triton X-100 (Caledon; Georgetown, ON) PBS, blocked with 10% horse serum in 0.1% Triton X-100 PBS, and incubated with fibronectin (ab23750; Abcam; 1:100) primary antibody for 8–12 hours at 4 °C. Primary antibodies were detected using Cy5-conjugated anti-rabbit (Molecular Probes; Carlsbad, CA; 1:200). All sections were counterstained with Hoechst 33342 dye (1:5000) for nuclei. Images were taken on Carl Zeiss Imager M2m microscope (Carl Zeiss; Jena) using ZenPro 2012 software.

### RT-qPCR

Gingival tissues were dissected out from the rats 3, 7 and 14 days post-wounding, and HGFs were cultured on collagen type I and rhPN pre-coated plates as previously described for 1 day and 7 days. Total RNA was isolated using 1 ml of TRIzol® reagent (Ambion; Carlsbad, CA, USA) per well according to the manufacturer’s recommendations. Real-time quantitative polymerase chain reaction (RT-qPCR) was performed on 50 ng of total RNA using TaqMan qScript^TM^ One-Step qRT-PCR Kit (Quanta; Gaithersburg, MD, USA) and gene-specific TaqMan probes (Applied Biosystems; Carlsbad, CA, USA) under following conditions: 48 °C for 30 minutes followed by 90 °C for 10 minutes and 40 cycles of 95 °C for 9 seconds and 60 °C for 1 minute using 7900 Real Time PCR system (Applied Biosystems). *Postn*, *Acta2*, *Col1A2*, *Col3A1*, *Fn1*, *Lox*, *POSTN*, *ACTA2*, *COL1A2*, *COL3A1*, and *FN1* mRNA expressions were normalized to the housekeeping gene, *18 S*. PCR efficiency was verified by dilution series and relative mRNA levels were calculated using the ΔΔCT method^66^.

### RT-PCR

Periostin isoforms were investigated by reversed transcriptase polymerase chain reaction (RT-PCR). Total RNA was isolated using 1 ml of TRIzol® reagent per well according to the manufacturer’s recommendations. 500 ng of mRNA was reverse transcribed using qScript™ cDNA SuperMix (Quanta Biosciences) following the manufacture’s protocols. cDNA was subjected to a 10 μL of PCR reaction mixture using a thermo cycler for 40 cycles. Following an initial denaturation of 6 minutes at 95 °C, PCR reaction was conducted for 40 cycles that consisted of 40 s at 94 °C for denaturation, 30 s at 43 °C for annealing, and 40 s at 72 °C for extension. The amplification of periostin cDNA was accomplished using a primer pair spanning exons 16 and 23 (Ensembl Exon accession numbers for rat: ENSRNOE00000121871 and ENSRNOE00000340025). PCR products were run on 1% agarose gels and detected with ethidium bromide.

### HGF isolation and growth

Healthy human gingival fibroblasts (HGFs) were isolated from tissue using the explant culture method^67^. All tissues were collected under informed consent from patients and with approval from the Western University Health Science Research Ethics Board. Isolation and experimental use of HGFs from human tissue were approved by the Western University Review Board for Health Sciences Research Involving Human Subjects and are in accordance with the 1964 Declaration of Helsinki. HGFs were maintained in high glucose Dulbecco’s modified Eagle’s medium (DMEM) (Gibco; Carlsbad, CA) supplemented with 10% fetal bovine serum (FBS; Gibco) and 1X antibiotics and antimycotics (AA; 100 μg/ml penicillin G, 50 μg/ml gentamicin, 25 μg/ml amphotericin B; Gibco), in 75 cm^2^ tissue culture plastic flasks, at 37 °C in a humidified atmosphere of 5% CO_2_. Cells were removed from the growth surface with trypsin-EDTA (0.05%; Gibco). Cells between passage 3 and 7 were used in experiments.

### Recombinant Human Periostin Plate Coating

Tissue culture plates were coated overnight at 4**°**C with 10 μg/ml bovine collagen type I (Advanced BioMatrix; San Diego, CA), or a combination of bovine collagen type I and 10 μg/ml recombinant human periostin (rhPN) (R&D Systems; Minneapolis, MN). Plates were then blocked with 1% bovine serum albumin at 37 **°**C for 1 hours.

### Cell treatment

HGFs, previously serum-starved in low glucose DMEM for 12–16 hours, were suspended in DMEM (high glucose) supplemented with 1X antibiotics/antimycotic (Gibco), 0.5% FBS (Gibco), and 50 μg/ml L-ascorbic acid (Sigma Aldrich), and were then seeded at 7000 cells per cm^2^ surface area in 24 well plates for proliferation assay experiment, and in 6 well plates for RT-qPCR, hydroxyproline assay, and western blot experiments. For assessing the influence of pathway inhibition, starved HGFs were independently pretreated with PF-573,228 (10 μg/ml), U0126 (10 μg/ml), and SP600125 (50 μg/ml) 30 minutes prior to seeding the cells on the coated plates as previously described. DMSO served as a control for PF-573,228 and SP600125, and U0124 (10 μg/ml) was used as a control for U0126. While cells were maintained at 37°C in a humidified atmosphere of 5% CO_2_, 50% of the conditioned media was changed every 48 hours with freshly prepared DMEM.

### CyQUANT® Proliferation Assay

After HGFs were cultured on collagen type I and rhPN pre-coated plates as previously described for 1, 3, 5, 7, and 9 days, media was completely aspirated and the plate was frozen at −80 °C. Once all time-points were captured, DNA contents were determined by performing CyQUANT® Cell Proliferation Assay Kit (C7026; Molecular Probes). Cell numbers were extrapolated using a standard curve.

### Hydroxyproline Assay

HGFs were cultured on collagen type I and rhPN pre-coated plates as previously described for 14 days. HGFs cultured with 5 ng/ml of TGF-β1 (R&D Systems) on collagen alone-coated plates were used as a positive control. After 14 days of culture, cell lysates and supernatants were obtained and hydroxyproline assay was performed in accordance with Hydroxyproline Assay Kit (MAK008; Sigma Aldrich). In brief, cell lysates and supernatants were hydrolyzed in 6 M HCl at 95 °C overnight. Oxidized hydroxyproline reacted with 4-(Dimethylamino)benaldehyde and resulting colorimetric (560 nm) product was read on the plate reader (Tecan Safire; Seestrasse, Männedorf). Hydroxyproline levels were extrapolated using a standard curve. In parallel cultures, cell numbers were quantified using CyQUANT® assay, as described, to use to normalize the corresponding hydroxyproline contents in each condition.

### Western Blotting

HGFs were cultured on collagen type I and rhPN pre-coated plates as previously described for 1 and 7 days. Western blotting was performed as previously described^68,69^. In brief, proteins were harvested with RIPA buffer (Sigma Aldrich) containing protease and phosphatase inhibitor cocktails. Protein concentration was determined by Pierce^®^ BCA Protein assay kit (Pierce; Waltham, MA). 25 μg proteins of each sample were separated by sodium dodecyl sulfate polyacrylamide gel electrophoresis (SDS-PAGE) and transferred to nitrocellulose membranes. Membranes were washed with Tris-buffered saline containing 0.05% Tween-20 (TBS-T) and blocked with 5% dried milk in TBS-T. Primary antibodies for fibronectin (sc-8422; Santa Cruz Biotechnology; 1:1000), α-SMA (A5228, Sigma Aldrich, 1:1000), and GAPDH (MAB374; Millipore; 1:2000) were used to incubate the membranes for 12 hours. Detection was with appropriate peroxidase-conjugated secondary antibodies (Jackson ImmunoResearch; West Grove, PA; 1:2000), which were developed with Clarity Western ECL substrate (Bio-Rad; Hercules, CA). Densitometry analysis was performed using ImageJ software version 10.2 (National Institutes of Health; Bethesda, MD).

### Immunocytochemistry

HGFs were cultured on collagen type I and rhPN pre-coated plates for 7 days. Cells were fixed with 4% paraformaldehyde, permeabilized with 0.1% Triton X-100 and blocked with 1% BSA (Thermo Fisher Scientific). Fixed and permeabilized cells were labeled with mouse anti-α-SMA (A5228, Sigma Aldrich; 1:400), which was detected with anti-mouse IgG conjugated to Alexa Fluor 488 secondary antibody (Molecular Probes; 1:200). α-SMA was double immunolabeled with rhodamine-conjugated phalloidin (Molecular Probes; 1:100) for filamentous actin. Nuclei were counterstained using Hoechst 33342 dye (1:5000). Images were taken on Carl Zeiss Observer Z1 microscope using AxioVision Relative software.

### Fixed Gel Contraction Assay

*In vitro*, contractility of HGFs was evaluated by employing collagen gel matrix contraction assays as previously described^70^. HGFs suspended in 0.5% FBS DMEM were mixed 1:1 with collagen mix [10% 0.2 M HEPES buffer (4-(2-hydroxyethyl)−1-piperazineethanesulfonic acid; pH = 8), 40% bovine collagen type I (Advanced BioMatrix), and 50% 2X high glucose DMEM (Gibco)] to a final density of 100,000 cells/ml. In parallel, either 5 μg/ml rhPN (R&D Systems) or an equivalent volume of PBS was incorporated into the collagen and cell mix. 24 well tissue culture plates were pre-coated with 1% BSA for 12 hours and washed with PBS. 1 ml of the cell and collagen mix was plated to each well and allowed to set at 37 °C. Following polymerization, 1 ml of 0.5% FBS DMEM was added to the wells. After 24 hours, the gels were detached from the plate and they were left to contract for 24 hours at 37 °C. As contraction of the collagen matrix excluded growth medium, thereby reducing the gel weight^71^, loss of gel weight was used to measure the extent of contraction. This accounted for contraction of gels horizontally and vertically.

### Adhesion assay

In parallel cultures, 25,000 cells/ml of HGFs were neutralized independently with 10 μg/ml integrin subunit specific anti-β1 (MAB2253; Millipore), and anti-αvβ3 (CBL544, Millipore), and anti-αvβ5 (MAB1961, Millipore) and control IgG antibody for 30 minutes with gentle agitation prior to seeding. 20,000 cells was cultured for 1 h on 24 well plates coated with collagen alone or collagen with rhPN. Unattached cells in the media were removed and attached cells were rinsed with PBS. Number of bound HGFs were determined using CyQuant® Assay (Molecular Probes) experiments were done in triplicates and 3 independent experiments.

### Enzyme-linked immunosorbent assay (ELISA)

HGFs were cultured on collagen and collagen + rhPN in serum free media, with media sampled at 3, 12 and 24 hrs. The concentrations of TGF-β1 in the cell culture conditioned media were quantified by Human TGF-β1 Quantikine ELISA System (R& D Systems). 100 μl of conditioned media and TGF-β1 standards were immunoassayed according to the manufacture’s protocol.

### Statistical Analysis

All statistical analysis was performed using Graphpad Software version 6 (Graphpad Software; La Jolla, CA) (*p* < 0.05 was considered significant). For *in vitro* study, data are expressed as the mean ± standard deviation of three individual experiments with independent primary cultures from different subjects. Individual experiments included three replicates. For RT-qPCR, western blot densitometry, gel contraction, and proliferation assays to assess the influence of rhPN, statistical analysis by Student’s t-test (unpaired) was performed. To compare hydroxyproline contents, one-way ANOVA with Bonferroni multiple comparisons test was used. For western blot densitometry of inhibition studies and adhesion assay, two-way ANOVA with Bonferroni multiple comparisons test was used.

For *in vivo* study, RT-qPCR data are expressed as the mean number ± standard deviation of six rats from independent litters. For PCNA^+^ cell quantification in the epithelium *in vivo*, data are expressed as the mean number normalized to the corresponding length of re-epithelialization ± standard deviation of three rats from independent litters. Statistical analysis was performed using  one-way ANOVA with Bonferroni multiple comparisons test.

## Electronic supplementary material

Supplementary figures 1, 2, 3

## Data Availability

The authors agree to make materials, data and associated protocols promptly available to readers without undue qualifications in material transfer agreements.
