# Supplementary material for: Fibronectin synthesis, but not α-smooth muscle expression, is regulated by periostin in gingival healing through FAK/JNK signaling
Source: Sci Rep. 2019 Feb 25;9:2708. doi: 10.1038/s41598-018-35805-6 (PMC6389918; doi:10.1038/s41598-018-35805-6)

Fibronectin synthesis, but not α-smooth muscle expression, is regulated by periostin in gingival healing through FAK/JNK signaling

Shawna S. Kim[1, 4], Georgia E. Nikoloudaki[1], Sarah Michelsons[1], Kendal Creber[2], and Douglas W. Hamilton[1, 2, 3, 4]*

[1]Department of Anatomy & Cell Biology,
[2]Department of Biomedical Engineering,
[3]Division of Oral Biology,
[4]Faculty of Dentistry,
Schulich School of Medicine and Dentistry, The University of Western Ontario
London, Ontario, Canada N6A 5C1

Supplementary files

Supplementary material Fig. 1. Gingivectomy in a rat model.

Gingivectomy was performed in adult female Wistar rats. Soft gingival tissue was removed. **A**. Diagram of posterior view of palate of the rat. Dark red rectangle (**arrowheads**) denotes where the gingivectomy was performed. **B**. Image of gingival wound, indicated by **arrowheads**, in our rat model. **C**. Histological staining (Masson's trichrome) of gingival wound from rats sacrificed right after the creation of the wound (**arrowheads**).

Supplementary material Fig. 2. HGFs cultured with rhPN exhibit increased collagen synthesis.

A. HGFs cultured on collagen or collagen + rhPN coated plates for 1 day and 7 days were assessed for gene expressions of *COL1A2* and *COL3A1*. Target gene expression was normalized to *18S* using the ΔΔCt method. Data represents mean fold gene expressions ± s.d. relative to control day 1 (collagen alone) of 3 independent experiments in triplicates. Data was analyzed via Student's t-test (unpaired) within each time-point (*p<0.05; ns, not significant). **B**. HGFs cultured on collagen alone or collagen + rhPN coated plates for 14 days were assessed for hydroxyproline contents in cell lysates and supernatant obtained at 14 days. HGFs cultured with TGF-β1 on collagen alone-coated plates were used as a positive control. Data represents mean hydroxyproline contents ± s.d. relative to control (on collagen alone) of 3 independent experiments in triplicates. Data was analyzed with one-way ANOVA (*p<0.05, **p <0.01, ***P<0.001, ****P<0.0001).

Supplementary material Fig. 3. Primary delete control for periostin labeling.

Sections were incubated without the primary antibody to periostin. Following this, ImmPRESS Reagent Kit Peroxidase (Vector Laboratory; Burlingame, CA) and DAB reagent (Vector Laboratory) were performed on the sections. Images demonstrate that the secondary antibodies did not bind to the tissue in the absence of the periostin primary antibody. Scale bar = 50 μm.

Supplementary Figure 1

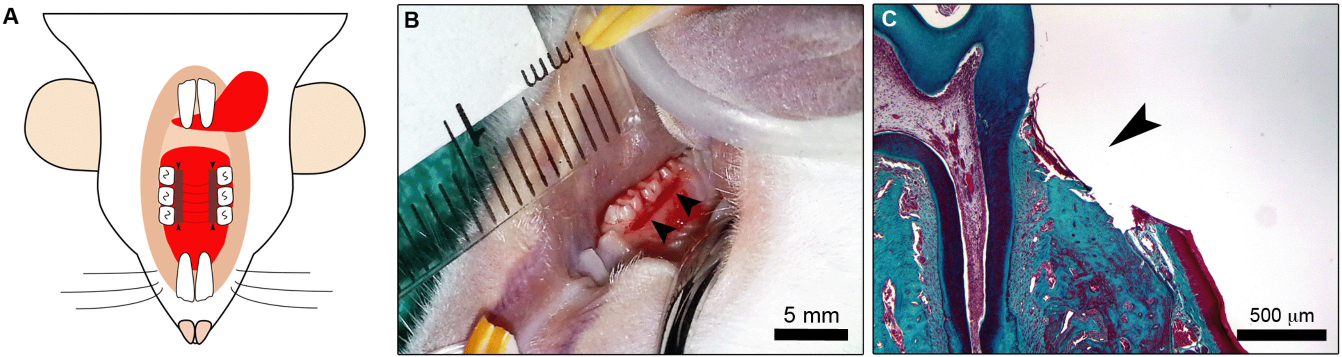

Supplementary Figure 2

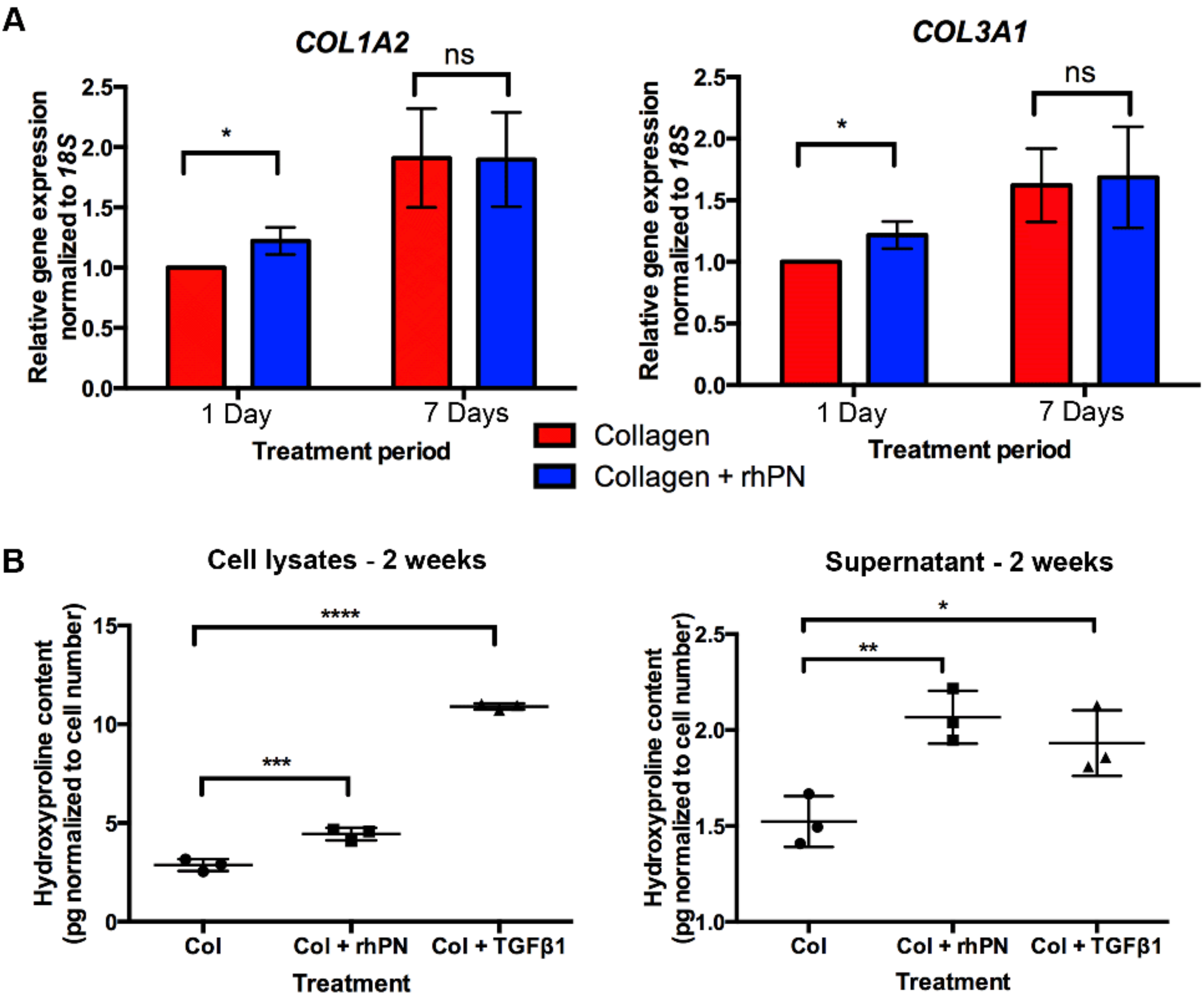

Supplementary Figure 3

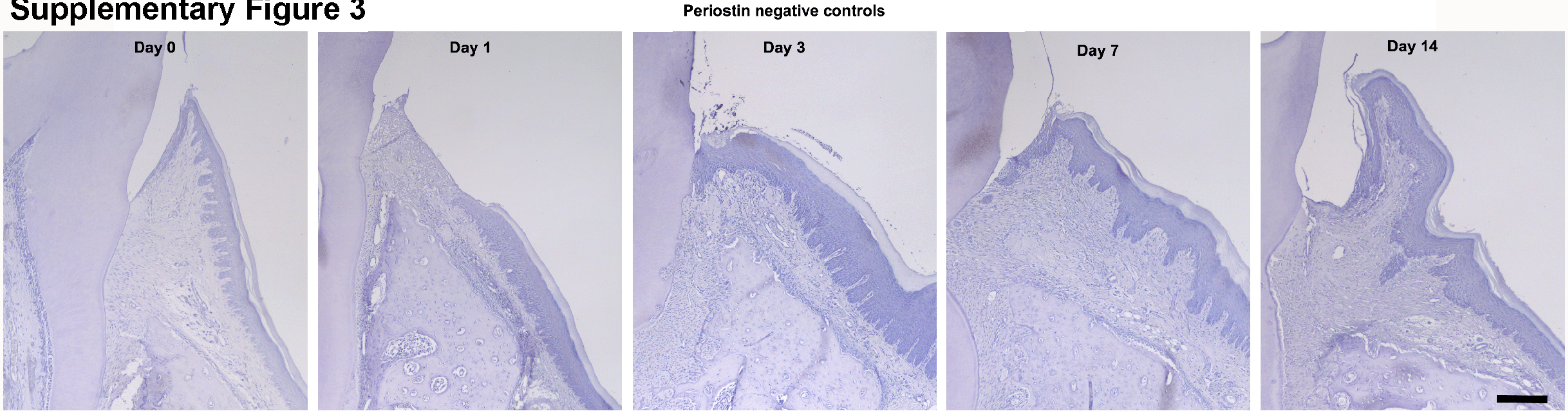

Supplement: Supplementary file 1 — Supplementary figures 1, 2, 3 [file 41598_2018_35805_MOESM1_ESM.pdf]
